# Retrieval tasks differentially influence memory for object features

**DOI:** 10.3389/fcogn.2026.1958570

**Published:** 2026-09-30

**Authors:** Katherine Archer, Kimele Persaud

**Affiliations:** Department of Psychology, Rutgers University, Newark, NJ, United States

**Keywords:** long-term memory, object-feature representations, recognition memory, retrieval effects, visual memory

## Abstract

Visual memory for objects and their constituent features (e.g., size, color, and state) is highly malleable and heavily influenced by encoding contexts and specific task-based characteristics. Yet, how object-feature memory is influenced by different retrieval tasks is not well understood. In this study, we examined whether variations in retrieval tasks that have been previously used in the literature (paired AFCs vs. 2-AFC) could produce different patterns of memory responses, that could then lead to different interpretations of the underlying memory representations. Specifically, participants studied and were asked to identify which object-feature combination they had previously seen. The results revealed that response patterns differed based on retrieval tasks, whereby the paired-AFC condition produced more accurate object-feature responses, while the 2-AFC produced more single-exemplar responses. Taken together, these results suggest that the nature of retrieval tasks can influence the kinds of elicited responses, leading to variation in interpretations across studies. Investigating how retrieval context shapes object-feature recognition provides important insight into malleability of visual object-feature representations in memory.

## 1 Introduction

Over the past few decades, the study of how objects and their constituent features are remembered has been the focus of much research. Substantial work has leveraged object-feature memory responses to shed light on the capacity and fidelity of memory (Bays et al., 2009; Lew et al., 2016; Persaud and Hemmer, 2016), the nature of the underlying object-feature representations (Ceraso et al., 1998; Balaban et al., 2020; Walker and Cuthbert, 1998; Utochkin and Brady, 2020), and the accuracy of eyewitness object memory (Loftus et al., 1978; Sarwar et al., 2014). Yet, object-feature memory is likely malleable and responses might be shaped by two critical factors: (1) differences in the encoding contexts in which objects are placed into memory (Castelhano and Henderson, 2005; Evans and Wolfe, 2022; Hollingworth and Henderson, 2002) and (2) variations in retrieval tasks that produce responses used to interpret the nature of underlying representations (Awh et al., 2007; Parks and Yonelinas, 2009; Tulving and Thomson, 1973; Voormann et al., 2021). While past research has examined how encoding contexts influence object-feature memory, far less work has explored how different retrieval tasks shape the kinds of responses individuals produce, specifically that inform our interpretation of underlying memory properties. To this end, this paper examines how variations in retrieval tasks influence object-feature responses and recognition accuracy. Investigating whether response patterns differ as a function of retrieval task is critically important for evaluating the correspondence between memory output and performance and underlying memory representations.

Extensive prior research has sought to leverage individual's object-feature (e.g., color, location, and orientation) memory responses to shed light on the capacity and fidelity of memory representations (Bays et al., 2009; Lew et al., 2016; Persaud and Hemmer, 2016; Persaud et al., 2025; Van den Berg et al., 2012; Zhang and Luck, 2008). Generally, in these studies, participants are tasked with recalling various features of objects. Yet, the retrieval tasks used across studies can vary between continuous report (e.g., Persaud and Hemmer, 2016; Van den Berg et al., 2012; Zhang and Luck, 2008), forced-choice recognition (Gloede and Gregg, 2019; Persaud et al., 2025), and change detection (e.g. Pashler, 1988; Wheeler and Treisman, 2002; Wilken and Ma, 2004). Yet, little research has directly examined the degree to which object-feature memory responses may differ as a function of the types of retrieval tasks used to assess memory.

Object-feature memory responses have also been used to address the long-standing debate on the nature of stored memory representations. Early research posited that objects and features may be represented as bound units of information. This theory was supported by empirical work demonstrating that increasing the number of to-be-remembered objects impairs memory, but increasing the number of features does not (Luck and Vogel, 1997; Vogel et al., 2001). In contrast, there is also evidence for unbound representations, such that increasing the number of object features worsens memory for all features (Fougnie et al., 2010; Fougnie and Alvarez, 2011; Markov et al., 2021). Importantly, support for both theories hinges on object-feature recognition responses which are likely influenced by the nature of the retrieval task. Yet, little research has directly compared the pattern of responses produced from different retrieval tasks, particularly object-feature recognition responses that are used to interpret the boundedness of underlying representations.

A third domain where the interpretation of object-feature memory responses is critically important is eyewitness object memory. Indeed, prior eyewitness memory has examined how individuals recognize object features from memory including: the color and shape of traffic signs (Loftus et al., 1978), the color of cars (Sarwar et al., 2014), the state of car headlights (broken/unbroken—Loftus, 1975), and the presence or absence of a logo (Brown, 2003). Responses to these object-feature questions are then used to support hypotheses about how eyewitness memory is shaped by various factors. Yet, here too object-feature memory responses might be influenced by differences in retrieval tasks.

Taken together, the aforementioned domains of memory fidelity and capacity, the nature of stored representations, and eyewitness memory, (and likely others) all draw support for theories about underlying properties of memory from object-feature responses. Yet, studies within and between domains rely on varying retrieval tasks which could potentially shape the pattern of responses observed^1^. Thus, it is important to reconcile not only how memory is shaped by specific manipulations employed in extant research, but also how memory responses might be altered by retrieval tasks.

Empirical support for theoretical assumptions about the capacity and fidelity of memory, the nature of representations, and eyewitness memory traces, all hinge on the specific object-feature memory responses individuals produce. Since underlying representations cannot be directly examined, we must make inferences based on what information individuals retrieve or fail to retrieve given their responses at test. Past research has shown that performance can be heavily influenced by slight variations in the retrieval test (Brady et al., 2023; Makovski et al., 2010; Naveh-Benjamin and Kilb, 2012; Stanislaw, 1995; Yeshurun et al., 2008). Even within a task, research has shown that slight differences in design such as requiring people to make single vs. simultaneous decisions, can alter performance (Parks and Yonelinas, 2009; Voormann et al., 2021). While past work has found differences in recognition accuracy as a function of retrieval task, the mechanisms by which those differences persist were unclear.

Relatedly, eye-witness memory research has found that memory judgements can be influenced by whether information is presented simultaneously or sequentially (Finley et al., 2015, 2020; Meissner et al., 2005; Mickes et al., 2012; Steblay et al., 2011). While some research has found that simultaneous line-ups in the retrieval test can lead to higher false alarms compared to sequential line-ups (see Steblay et al., 2011), simultaneous presentation can also, in some contexts, produce overall superior recognition accuracy (Finley et al., 2015; Wixted and Mickes, 2014). The advantage for simultaneous presentation is attributed to heightened discriminability between presented stimuli allowing observers to better isolate the necessary distinctive features between options to facilitate more accurate recognition judgments (Finley et al., 2015; Wixted and Mickes, 2014). Given that memory is highly sensitive to the retrieval task, this poses a potential problem for interpreting how information is represented based on responses obtained during retrieval. Work investigating object-feature representations relies on the responses individuals give during retrieval and the kinds of errors made to infer how that information was stored in long-term memory. While responses may vary across tasks, leading to differences in interpretation, the underlying representations likely remain unchanged. Thus, it is important to determine whether differences in object-feature recognition performance across retrieval tasks reflect differences in memory sensitivity or arise from decision bias (e.g., strategic guessing). Although this distinction has been examined in other domains (Finley et al., 2015; Wixted and Mickes, 2014), it has not yet been examined in the context of object-feature recognition.

In the current study, we sought to investigate whether variations in retrieval tasks impact the pattern of memory responses participants produced. In the task, individuals studied two exemplars from the same object category (e.g., two different mugs) in either the same or different state (e.g., empty vs. full). To assess the role of retrieval tasks, two tasks were employed: a traditional n-AFC and a paired 2-AFC where two decisions are made on each trial. These two tasks have been used in prior research (paired 2-AFC: Utochkin and Brady, 2020, traditional n-AFC: Balaban et al., 2020) with each task providing support for different types of underlying memory representations.^2^ While the nature of the retrieval test was not the sole difference between the two aforementioned studies, Balaban et al. (2020) emphasized that different performance patterns can be elicited based on how the retrieval test is designed. Thus, in the current study, we examine whether simultaneous (paired 2-afc) vs. sequential testing (traditional 2-afc) within a two-exemplar design, can produce differences in recognition responses and response dependence.

We hypothesized that responses in the paired 2-AFC task might not be independent, such that correct identification of one exemplar may be linked to responses for the other exemplar when presented simultaneously. Since the paired design presents additional information during retrieval (two targets and two foils), we suspect that the discriminability advantage observed for simultaneous presentations (Finley et al., 2015) might be amplified in this condition, increasing overall accuracy. In contrast, the traditional 2-AFC may be more independent as judgments are not made at the same time. As such, we would expect differences in responses between the two tasks that might ultimately influence how underlying representations are interpreted. Finally, we also included a time manipulation to assess whether differences in encoding time might also impact object-feature binding.

## 2 Method

### 2.1 Participants

Eighty Eight undergraduates (Mage = 20.35 years^3^, SD = 4.83; 71% Female) at Rutgers University—Newark participated in this study for course credit. Two participants were excluded for missing more than 40% of test responses, and one participant for reporting non-normal color vision. The remaining missing trials were dropped from analyses. Remaining participants self-reported normal or corrected-to-normal vision and normal color vision. Written informed consent was obtained from all participants prior to the start of the study and procedures were approved by the Rutgers University Institutional Review Board. A total of 85 subjects were included in the final analyses (2-AFC-1s: *n* = 21; 2-AFC-3s: *n* = 21; Paired-AFC-1s: *n* = 20; Paired-AFC-3s: *n* = 23). A sensitivity analysis conducted in G^*^Power (v3.1—Faul et al., 2007) indicated that, with an alpha level adjusted for multiple comparisons (α = 0.0125), a sample size of *N* = 85 provided 80% power to detect an interaction effect as small as Cohen's *f* = 0.179 between retrieval task and trial type.

### 2.2 Stimuli and apparatus

The stimuli consisted of 72 discrete object categories (e.g., mug), with two different exemplars (e.g., Mug 1, Mug 2) presented in two different states (e.g., full, empty) for each category (see Figure 1A). Images were licensed through Adobe Stock with a subset of images selected from the objects used in Brady et al. (2013a). There were a total of 288 unique exemplar-state images in the full stimulus set. Each participant viewed half of the objects during study, while the remaining objects were reserved for the test phase. Exemplar-state combinations were randomized across subjects. The experiment was programmed in Matlab version R2019b (MathWorks Inc, 2024) and presented on a Windows computer with an 11th Gen Intel(R) Core(™) i5-11500 processor (75 Hz at 1920 x 1080 resolution). The entire study took approximately 30 min.

**Figure 1:**
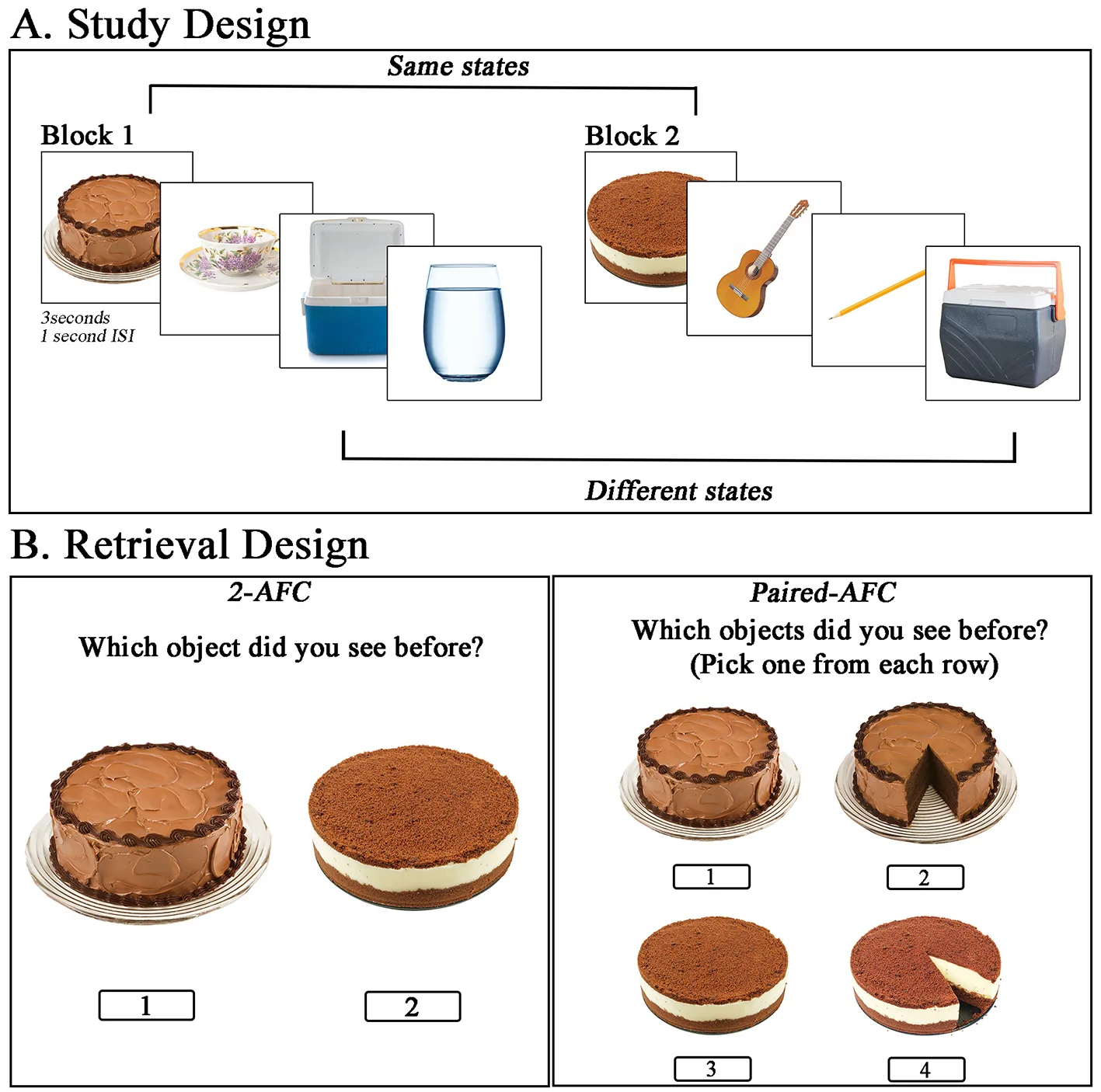
Study **(A)** and test **(B)** procedures. **(A)** Objects appeared in either the same state or different states across blocks. **(B)** Objects were presented two at a time, with one previously seen object and one novel object (2-AFC) or four at a time with two previously seen objects and two novel objects (Paired-AFC). Stimuli pictured above were adapted from images licensed through Adobe Stock.

### 2.3 Design and analysis

The study employed a 2 (Between subjects—Retrieval Task: Paired-AFC, 2-AFC) x 2 (Between Subjects—Study Time: 1-s, 3-s) x 2 (Within subjects—Trial Type: different states, same states) mixed design. Participants were randomly assigned to complete either a traditional 2-Alternative Forced Choice (AFC) recognition task or a paired 2-AFC. In the paired 2-AFC, exemplars were paired by object category (e.g., mug) and participants were shown both exemplars in the state they had studied them and a new state (see Figure 1B). On each trial participants were asked to make two simultaneous choices about which two exemplars they had studied. Object presentation time was also randomly assigned. Across blocks, exemplars either appeared in the same state (e.g., both full) or different states (e.g., full, empty). To avoid systematic guessing, there were an equal number of trials. Trial type for each exemplar was counterbalanced across four sets, and sets were randomized across participants.

Overall accuracy was calculated as the proportion of correct responses across all trials. Paired accuracy was calculated by taking the proportion of responses for each object category where both exemplars were correctly identified, one exemplar was correctly identified, or neither exemplar was correctly identified. Initial analyses revealed no significant differences based on presentation time, as such all subsequent analyses were collapsed across presentation time. A series of 2 (Retrieval Task) x 2 (Trial Type) mixed ANOVAs were conducted, one for each of the four response types (Overall Accuracy, Both Correct, One Correct, and None Correct).

To assess whether responses for each exemplar were independent of each other, we ran a mixed-effects logistic regression. If responses are independent, accuracy for the first exemplar should not predict accuracy for the second exemplar. Fixed effects included first exemplar accuracy (reference: correct), retrieval task (reference: 2-AFC), and trial type (reference: different trials), and all two- and three-way interactions. We included random intercepts for subjects to account for individual differences in overall performance. We first compared a reduced model including all main effects and two-way interactions to a full model including a three-way interaction between exemplar 1 accuracy, retrieval task, and trial type. The full model provided a significantly better fit than the reduced model, χ^2^(1) = 27.73, *p* < 0.001.

Analyses were conducted in Python (Python Version 3.12) and *R* (Version 4.3.3; R Core Team, 2024). All data was normally distributed and passed tests of homogeneity of variance. The Holm correction was applied to all pairwise comparisons to adjust for multiple comparisons (Holm, 1979). Data and analysis code are available on OSF. This study was not pre-registered.

### 2.4 Procedure

The experiment consisted of a study and test phase. The study phase was divided into two blocks to ensure separation of the two exemplars; in each block 72 objects were presented in isolation on a white background. Objects were presented in the center of the screen for either 1-s or 3-s, followed by a 1-s fixation cross. One exemplar appeared in each block, while across blocks exemplars were presented in either the same or different states (see Figure 1A). Participants were instructed to study the object as their memory would be tested later. Importantly, participants were not informed of the type of retrieval test that would be used. Further, memory was only tested after both blocks were completed.

In the test phase, participants either completed the traditional 2-AFC or a paired 2-AFC. Participants in the 2-AFC condition were presented with only one exemplar on each trial and asked to choose which object they remember (objects differed only by state). Memory for the second exemplar was tested on a later trial. In the paired 2-AFC, objects were presented in a 2x2 array where each row contained both state images for one exemplar (one they had previously studied and one new image). A response box appeared to the right of each row, for each row participants entered the number corresponding to the image they remembered studying. Informed consent was obtained for all participants upon their arrival, followed by a short demographic questionnaire (Qualtrics, 2024).

## 3 Results

### 3.1 Presentation time

We first evaluated whether recognition performance differed based on presentation time (i.e., 1s vs. 3s). A 2 (Retrieval Task: 2-AFC or Paired-AFC) x 2 (Presentation Time: 3s or 1s) ANOVA revealed no significant main effect of time, *F*(1,166) = 0.13, *p* = 0.72, η_*p*_^2^ = 0.001, or task, *F*(1,166) = 2.79, *p* = 0.10, η_*p*_^2^ = 0.017, and no interaction, *F*(1,166) = 2.94, *p* = 0.09, η_*p*_^2^ = 0.017. Since presentation time had no effect, all subsequent analyses collapsed across presentation time.

### 3.2 Overall accuracy

Next, we examined whether overall accuracy differed by retrieval task and trial type (see Table 1). A 2 x 2 mixed ANOVA revealed no significant main effect of task, *F*(1,83) = 1.72, *p* = 0.19, η_*p*_^2^ = 0.020, but a significant main effect of trial type, *F*(1,83) = 42.47, *p* < 0.001, η_*p*_^2^ = 0.339. Participants correctly identified more exemplars presented in the same state (*M* = 0.81, *SD* = 0.11) than different states (*M* = 0.76, *SD* = 0.12), *t*(84) = 5.57, *p* < 0.001, Cohen's *d* = 0.483. There was also a significant interaction, *F*(1,83) = 32.08, *p* < 0.001, η_*p*_^2^ = 0.279. *Post-hoc* comparisons revealed that for different states, participants in the paired-AFC condition correctly identified more exemplars (*M* = 0.79, *SD* = 0.12) than those in the 2-AFC condition (*M* = 0.72, *SD* = 0.11), *t*(82.29) = 3.16, *p* < 0.01, Cohen's *d* = 0.687.

**Table 1:** Proportion correct by condition and trial type.

Condition	Trial type	Overall accuracy	Both correct	One correct	None correct
**2AFC**	**Different**	0.72 (0.11)	0.51 (0.18)	0.43 (0.16)	0.07 (0.06)
	**Same**	0.82 (0.11)	0.70 (0.16)	0.24 (0.12)	0.06 (0.06)
**Paired AFC**	**Different**	0.79 (0.12)	0.66 (0.19)	0.28 (0.15)	0.07 (0.06)
	**Same**	0.80 (0.11)	0.66 (0.18)	0.29 (0.15)	0.06 (0.05)

Overall accuracy is the proportion of all trials that were correct. Trials were then paired by category to obtain the mean proportion of paired trials where both exemplars were correct, only one exemplar was correct, or neither exemplar was correct. Mean and standard deviations (in parantheses) provided for all accuracy measures.

### 3.3 Accuracy for paired trials

We also assessed paired accuracy where exemplars were paired by object category (e.g., mug, trashcan) and marked as both correct (i.e., both exemplars identified in correct state), one correct (i.e., only one exemplar identified in correct state), or none correct (i.e., neither exemplar identified in correct state). If the retrieval test indeed differentially influences the pattern of memory responses participants make, we would expect different rates at which participants get both, one, or none correct (see Figure 2A).

**Figure 2:**
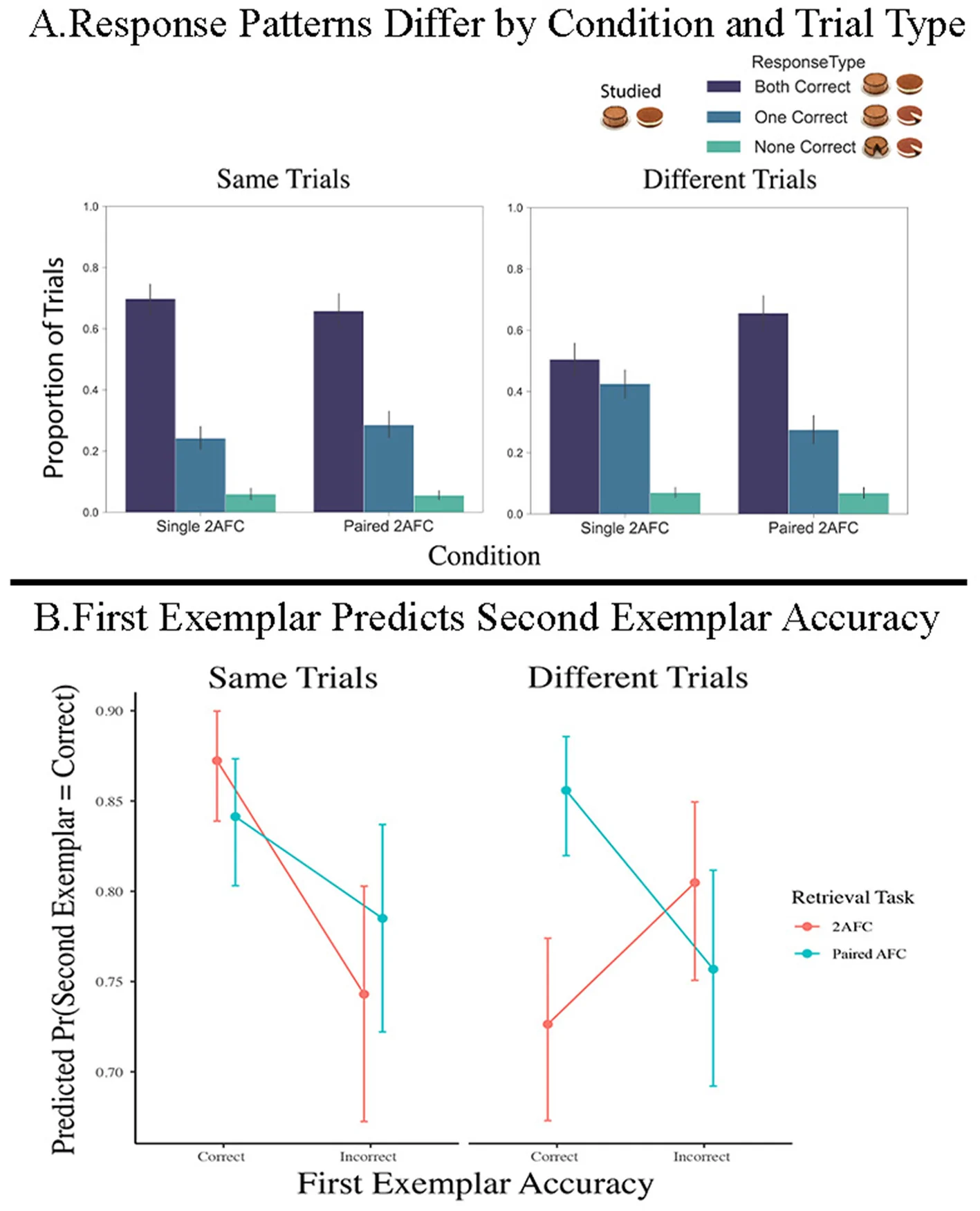
**(A)** Proportion of paired trials where participants correctly identified both exemplars (purple), only one exemplar (blue), or neither exemplar (green) for trials where both exemplars appeared in the same state (Left) or different states (Right). **(B)** The probability of correctly identifying the second exemplar based on accuracy for the first exemplar, retrieval task (Paired-AFC vs. 2-AFC), and trial type (same vs. different). Bars represent 95% CI.

#### 3.3.1 Both correct

A 2 x 2 mixed ANOVA revealed no significant main effect of retrieval task, *F*(1,83) = 2.50, *p* = 0.12, η_*p*_^2^ = 0.029, but a main effect of trial type, *F*(1,83) = 41.42, *p* < 0.001, η_*p*_^2^ = 0.333. Participants remembered both exemplars presented in the same state significantly more than (*M* = 0.68, *SD* = 0.17) exemplars in different states (*M* = 0.58, *SD* = 0.20). There was also a significant interaction, *F*(1,83) = 40.37, *p* < 0.01, η_*p*_^2^ = 0.327. When exemplars appeared in different states, participants who completed the paired-AFC task (*M* = 0.66, *SD* = 0.19) identified both exemplars in the correct state significantly more often than participants who completed the 2-AFC task (*M* = 0.51, *SD* = 0.18), *t*(82.84) = 3.84, *p* < 0.001, Cohen's *d* = 0.832.

#### 3.3.2 None correct

A 2 x 2 mixed ANOVA revealed no significant main effect of retrieval task, *F*(1,83) = 0.05, *p* = 0.82, η_*p*_^2^ = 0.0006, but a significant main effect of trial type, *F*(1,83) = 4.66, *p* = 0.03, η_*p*_^2^ = 0.05. Participants remembered neither exemplar significantly more when presented in different states (*M* = 0.07, *SD* = 0.06), compared to the same state (*M* = 0.06, *SD* = 0.06), and no significant interaction, *F*(1,83) = 0.05, *p* = 0.83, η_*p*_^2^ = 0.0006.

#### 3.3.3 One correct

A 2 x 2 mixed ANOVA revealed a marginal effect of task, *F*(1,83) = 3.73, *p* = 0.057, η_*p*_^2^ = 0.043. Participants were slightly better at correctly identifying one exemplar in the 2-AFC (*M* = 0.33, *SD* = 0.17) compared to the paired-AFC (*M* = 0.28, *SD* = 0.15) condition. There was a significant main effect of trial type but this time in the opposite direction, *F*(1,83) = 30.84, *p* < 0.001, η_*p*_^2^ = 0.271. Participants remembered only one exemplar more when presented in different states (*M* = 0.35, *SD* = 0.17) than the same state (*M* = 0.26, *SD* = 0.14). There was also a significant interaction, *F*(1,83) = 39.95, *p* < 0.001, η_*p*_^2^ = 0.325. On different trials, participants in the 2-AFC condition (*M* = 0.43, *SD* = 0.16) were more likely to identify only one exemplar in the correct state than those in the paired-AFC condition (*M* = 0.28, *SD* = 0.15), *t*(82.89) = 4.50, *p* < 0.001, Cohen's *d* = 0.977. This is likely due to participants getting both exemplars correct more often in the paired-AFC condition.

### 3.4 Response dependence

Since both exemplar states were correctly identified more often in the paired-AFC task, we assessed whether the dependence of responses differed by retrieval task. If responses were dependent, correctly identifying one exemplar should predict the probability of correctly identifying the second exemplar. Further if the retrieval task does not influence the kinds of responses individuals make, the pattern should not differ across the two tasks.

A linear mixed effects model revealed a significant three-way interaction between first exemplar accuracy, retrieval task, and trial type, (β = 1.57, *SE* = 0.30, *z* = 5.29, *p* < 0.001, OR = 4.81), indicating that the influence of first exemplar accuracy on second exemplar accuracy depended jointly on retrieval task and trial type (see Figure 2B). On different trials, in the paired-AFC condition, correctly identifying the first exemplar was associated with higher odds of correctly identifying the second exemplar. Conversely, in the 2-AFC condition, correctly identifying the first exemplar was associated with lower odds of correctly identifying the second exemplar. On same trials, in both the paired-AFC and 2-AFC conditions, correctly identifying the first exemplar was associated with higher odds of correctly identifying the second exemplar compared to incorrectly identifying the first (see Supplementary materials for full results).

### 3.5 Sensitivity analysis

To address the possibility that the difference in responses between the paired-AFC and the 2-AFC reflect differences in retrieval strategy, we additionally assessed performance using 2-AFC *d'*, an SDT-based measure of sensitivity, calculated as *d'* = $\sqrt{2}$ *z(PC)* (Hautus et al., 2021). If the accuracy advantage observed for the paired-AFC over the 2-AFC was a result of strategic guessing instead of better sensitivity, we would expect no difference in *d'* between the two conditions. That is, the accuracy advantage for the paired-AFC condition would be attenuated when *d'* is used, as this measure prevents guessing from inflating accuracy. In contrast, if the paired-AFC produces greater sensitivity, then we would expect the accuracy advantage for the paired-AFC condition on different trials to remain even when a more sensitive measure is employed. A linear mixed-effects model treating *d'* as the outcome measure, Trial Type, Encoding Time, and Retrieval Task as fixed effects, and participant as a random effect, revealed a significant main effect of trial type (*F*(1, 82) = 31.23, *p* < 0.001, η_*p*_^2^ = 0.28), no significant main effect of retrieval task (*F*(1,82) = 1.80, *p* = 0.186, η_*p*_^2^ = 0.02), and no significant effects involving timing (all *ps* > 0.05). However, there was a significant interaction between trial type and retrieval task (*F*(1,82) = 27.19, *p* < 0.001, η_*p*_^2^ = 0.25). Pairwise comparisons indicated that on different trials, sensitivity was significantly greater in the paired-AFC condition ($M_{d^{\prime }}$ = 1.29, *SE* = 0.09) compared to the 2-AFC condition ($M_{d^{\prime }}$ = 0.86, *SE* = 0.10), *t*(109) = 3.22, *p* < 0.01. Conversely, on same trials, there was no significant difference between paired-AFC condition ($M_{d^{\prime }}$ = 1.31, *SE* = 0.09) and the 2-AFC condition ($M_{d^{\prime }}$ = 1.41, *SE* = 0.10), *t*(109) = −0.75, *p* = 0.46). These results mirror the general accuracy and the “both correct” analyses, providing converging evidence that the retrieval format alters memory responses rather than promoting strategic guessing.

## 4 Discussion

In this paper, we sought to investigate whether the pattern of object-feature memory responses differed as a function of retrieval task. Past research has typically relied on memory responses during retrieval as a proxy for the underlying representation to inform theories about memory properties like fidelity and bound vs. unbound memory representations. One issue that arises is that memory is not always assessed in the same manner across studies which may lead to differences in responses (Brady et al., 2023; Finley et al., 2015; Makovski et al., 2010; Meissner et al., 2005; Mickes et al., 2012; Naveh-Benjamin and Kilb, 2012; Persaud et al., 2025; Stanislaw, 1995) and ultimately differences in interpretation. In the current study, we explored whether retrieval tasks produce different patterns of memory responses that in turn can influence the subsequent interpretation of underlying memory properties. We examined whether completing a paired-AFC or a traditional 2-AFC could produce differences in recognition accuracy and paired correct responses.

We found that slight variations in the retrieval task led to differences in the types of responses individuals produced. On trials where object-feature binding was more difficult (i.e., different trials), we found the paired-AFC condition produced more responses where both exemplar-feature pairs were correctly recognized. Comparatively, the 2-AFC produced significantly less exemplar-feature pairs (both correct), but more correct single exemplar-features (one correct). Since the encoding procedure was held constant across retrieval conditions, it is unlikely that the underlying memory trace differed, but rather that responses differed as a result of the type of retrieval task completed. Additionally, we did not find any difference in memory performance when encoding time varied. This could either reflect a true lack of difference between 1 and 3 s of study time or a lack of power to detect an encoding time effect in the current study. There is prior research that has found comparably object-feature memory, following 1 s and 3 s of time (Brady et al., 2013b). As such, it is possible that the addition of 2 s in our study did relatively little to improve already sufficient encoding with 1 s. Future research is needed to further explore encoding time effects on object-feature recognition accuracy.

Differences in responses only appeared on different trials, while performance on same trials was comparable across tasks, suggesting differences between retrieval tasks was related to individuals' ability to retrieve information that required object-feature binding. Showing individuals both exemplars simultaneously at test led to greater accuracy for both exemplars, suggesting that responses in the paired-AFC were not independent. Indeed, we found evidence that when participants completed the paired-AFC, response accuracy for one exemplar predicted greater accuracy for the other exemplar on different trials. Whereas when participants completed the 2-AFC, accuracy for the first exemplar predicted worse accuracy for the second exemplar. It is possible that during the paired-AFC individuals were able to leverage memory for one exemplar to guide responses for the other exemplar.

Our findings suggest that how information is retrieved can influence the types of memory responses produced as well as the accuracy of those responses. This is important because response accuracy and paired response judgments have been used to inform theories about the storage and nature of underlying memory representations. Retrieval tasks have been shown to interact with not only what information is retrieved but also the kinds of errors individuals make, such as in eyewitness memory studies (Finley et al., 2015, 2020; Meissner et al., 2005). The current study extends previous findings, with implications for the eye-witness memory literature, to research exploring object-feature representations in long-term memory. The current findings also lend support to the memory advantages associated with simultaneous presentation potentially owing to heightened discriminability between targets and foils. Indeed, we observed that presenting additional information during retrieval in the form of the paired AFC, produced higher recognition accuracy. Further, sensitivity analyses provided converging evidence that the retrieval format altered discriminability of exemplar-state pairings, rather than strategic guessing. Taken together, this study presents a call to action for carving out best practices for assessing memory, specifically for the purposes of making inferences about how object-feature representations are stored.

Additionally, this study opens up several interesting lines of future inquiry. For example, the current study calls into question how retrieval tests are designed. Simultaneous judgments lead to dependent responses while sequential judgments do not. Therefore, it is important to consider how this may influence interpretation when selecting the most appropriate design. More broadly, while this work explores differences in memory performance based on two different retrieval tasks, there are a host of other retrieval tests frequently used in the literature. Differences in the nature of those tests alone might contribute to variability in which object features individuals tend to prioritize in memory. As such, future work should examine whether using different tasks to assess the same underlying properties of memory promote inherent differences in performance that might not necessarily reflect the underlying representation.

In summary, differences in retrieval tasks can lead to differences in responses, which is the basis for our understanding of nearly every facet of memory (e.g., memory fidelity and capacity, long-term memory representations, and eyewitness memory). Given the evidence that the type of retrieval task can influence the kinds of responses individuals give during retrieval, future studies should explore best practices for investigating long-term memory representations that are less susceptible to study design as this often varies across studies.

## Data Availability

The datasets presented in this study can be found in online repositories. The names of the repository/repositories and accession number(s) can be found below: https://osf.io/gbs6n?view_only=311e34679b534b75b8abb271eaeccc0f.

## Footnotes

1 We do not mean to imply that differences between studies in these domains can be solely attributed to differences in retrieval tasks. Instead, we assert the retrieval test differences could contribute to some of the observed variability between studies. The goal of the current study is to assess whether this is the case using two different retrieval tasks that have been previously employed to study the same general memory construct (i.e., object-feature representations).

2 We do not make strict interpretations of whether the results reflect bound or unbound representations as these interpretations require ruling out alternative explanations (e.g., strategic guessing, individual responses strategies, etc.) that are beyond the scope of the current study.

3 Two participants were excluded from the age calculation due to a recording error.
