# Supplementary material for: Retrieval tasks differentially influence memory for object features
Source: Front Cognit. 2026 Sep 30;5:1958570. doi: 10.3389/fcogn.2026.1958570 (PMC13661476; doi:10.3389/fcogn.2026.1958570)
Supplement: Supplementary file 1 [file Table_1.docx]

**Supplementary Materials**

**Table 1**.

*Odds ratio from estimated marginal means contrasts examining the interaction between first exemplar accuracy, retrieval task, and trial type*

| **Retrieval Task** | **Trial Type** | **Comparison** | **OR** | **p** | **95% CI** | |
| --- | --- | --- | --- | --- | --- | --- |
| Paired-AFC | Different | Correct vs Incorrect | 1.91 | <.001 | 1.425 | 2.553 |
|  | Same | Correct vs Incorrect | 1.45 | 0.016 | 1.071 | 1.963 |
| 2AFC | Different | Correct vs Incorrect | 0.64 | <.001 | 0.492 | 0.843 |
|  | Same | Correct vs Incorrect | 2.38 | <.001 | 1.727 | 3.216 |

**Table 2.**

*Mixed Effects Logistic Regression on Second Exemplar Accuracy for First Exemplar Accuracy x Retrieval Task x Trial Type*

|  | ***b*** | | ***SE*** | | **z statistic** | | **Odds Ratio** | | **95% CI** | |
| --- | --- | --- | --- | --- | --- | --- | --- | --- | --- | --- |
| **1st Exemplar (Incorrect)** | | 0.44 | | 0.14 | | 3.21** | | 1.55 | | [1.19, 2.03] |
| **Retrieval Task (Paired-AFC)** | | 0.80 | | 0.19 | | 4.28*** | | 2.24 | | [1.55, 3.23] |
| **Trial Type (Same)** | | 0.94 | | 0.11 | | 8.51*** | | 2.55 | | [2.06, 3.17] |
| **1st Exemplar x Retrieval Task** | | -1.09 | | 0.20 | | -5.38*** | | 0.34 | | [0.23, 0.50] |
| **1st Exemplar x Trial Type** | | -1.30 | | 0.21 | | -6.23*** | | 0.27 | | [0.18, 0.41] |
| **Retrieval Task x Trial Type** | | -1.06 | | 0.16 | | -6.72*** | | 0.35 | | [0.26, 0.47] |
| **1st Exemplar x Retrieval Task x Trial Type** | | 1.57 | | 0.30 | | 5.29*** | | 4.81 | | [2.69, 8.62] |

*Note*. * p < .05 ** p < .01 *** p < .001; 95% confidence intervals are reported for odds ratios.
